# Placental malaria and its effect on pregnancy outcomes in Sudanese women from Blue Nile State

**DOI:** 10.1186/s12936-017-2028-0

**Published:** 2017-09-16

**Authors:** Samia A. Omer, Hagir E. Idress, Ishag Adam, Mutasim Abdelrahim, Ali N. Noureldein, Abdelrahim M. Abdelrazig, Mohammed O. Elhassan, Suad M. Sulaiman

**Affiliations:** 1 Department of Immunology and Biotechnology, Tropical Medicine Research Institute, National Centre for Research, Khartoum, Sudan; 2 School of Biomedical Sciences, Middlesex University, London, UK; 3 Faculty of Medicine, University of Khartoum, Khartoum, Sudan; 4 Ed-Damazin Hospital, Blue Nile State Ministry of Health, Ed-Damazin, Sudan; 5 Department of Microbiology, Faculty of Medicine, Blue Nile University, Ed-Damazin, Sudan; 6 Khartoum Teaching Eye Hospital, Khartoum State Ministry of Health, Khartoum, Sudan; 7 Sudanese National Academy of Sciences, Khartoum, Sudan

**Keywords:** Malaria during pregnancy, Placental malaria, Pregnancy outcomes, Blue Nile State, Sudan

## Abstract

**Background:**

Malaria infection during pregnancy can result in placental malaria and is associated with adverse pregnancy outcomes particularly among primigravidae. The aim of this study was to assess the prevalence and risk factors for placental malaria and its effect on pregnancy outcomes in Blue Nile state, Sudan.

**Methods:**

A cross-sectional hospital-based study was conducted consecutively during January 2012–December 2015 in three main hospitals in Blue Nile State, Sudan. At delivery, peripheral and placental blood samples were collected from consenting women. Finger prick blood was used for preparation of peripheral smears and for haemoglobin measurement. Smears were stained with Giemsa and examined microscopically for malaria parasites. Pregnancy outcomes in association to placental malaria were investigated.

**Results:**

A total of 1149 mothers and their newborns were recruited. The mean (SD) of the age was 23.3 (5.2) years. Detection of malaria parasites was confirmed in 37.8% of the peripheral blood films and 59.3% of the placental films with *Plasmodium falciparum* as the only species detected. In multivariate analysis, younger age ≤23.2 years old (AOR = 3.2, 95% CI 1.9–5.5; *P* < 0.001), primiparae (AOR = 3.9, CI 2.1–7.6; *P* < 0.001), secundiparae (AOR = 2.8, 95% CI 1.5–5.1; *P* < 0.001, no antenatal care (ANC) visits (AOR = 11.9, 95% CI 7.8–18.1; *P* < 0.001) and not using bed nets (AOR = 3.5, 95% CI 1.7–6.8; P < 0.001) were risk factors for placental malaria. Education and residence were not associated with placental malaria infection. Placental malaria was significantly associated with maternal anaemia (AOR = 41.6, 95% CI 23.3–74.4; P < 0.001) and low birth weight (LBW) (AOR = 25.2, 95% CI 15.1–41.3; *P* < 0.001).

**Conclusion:**

During the study, there was a high prevalence of placental malaria in Blue Nile State-Sudan, as the enhanced control activities were not practiced, leading to adverse pregnancy outcomes, such as maternal anaemia and LBW.

## Background

Malaria during pregnancy is a serious public health problem in sub-Saharan Africa and about 10,000 women and 200,000 babies die annually because of malaria in pregnancy. Most of these deaths are caused by *Plasmodium falciparum*, which is found in tropical and subtropical regions [1].

In malaria endemic areas, at least one in four pregnant women has evidence of peripheral or placental malaria at delivery [2]. It has been documented that malaria parasites may be detected in the placenta of women who are otherwise apparently uninfected; with a peripheral parasitaemia undetectable by microscopy [3, 4]. Moreover, women pregnant for the first time (primigravidae) are highly susceptible to malaria compared with multigravidae [5–7]. Malaria during pregnancy is characterized by the sequestration of infected erythrocytes (IEs) in placental inter-villous spaces binding chondroitin sulphate A (CSA) resulting in placental malaria (PM) [3, 8]. PM is associated with adverse pregnancy outcomes, such as miscarriage, stillbirth, preterm birth (PTB) [9, 10], low birth weight (LBW) and congenital malaria [4]. Congenital malaria occurs when the parasite crosses the placenta; leading to mother-to-child transmission of disease [11]. Moreover, the parasite invades large numbers of erythrocytes, causing severe maternal anaemia [9, 12].

In Sudan, malaria is one of the deadliest endemic diseases, and the increased susceptibility of pregnant women to malaria is a long-standing public health problem [6, 13].

Maternal mortality rate in Sudan in 2013 was 311/100,000 live birth [14]. The infant’s health is also at risk as a result of infection of the placenta and maternal anaemia caused by malaria. Both of these factors contribute to LBW [15], which is the leading cause of perinatal and infant mortality [16]. In Sudan, detailed data on the pattern and risk factors for placental malaria are scarce.

Primary health care in Blue Nile State is the worst in central Sudan in terms of health indicators accompanied by the role of migration due to political unrest. Infant and child mortality are higher than in other neighbouring states. Malaria is one of the major problems that increase mortality in the state with more than 30,000 cases reported in 2010 (a prevalence of 34%). Moreover, malaria is the main cause of common morbidity and mortality in the state [17]. In Sudan, there is no antenatal care programme to monitor coverage of intermittent preventive treatment (IPTp) using sulfadoxine–pyrimethamine (SP) to all pregnant women attending antenatal clinics as recommended in areas of high malaria transmission with low resistance to SP [18].

The current study was conducted to assess the prevalence and risk factors of placental malaria and its effect on pregnancy outcomes in Sudanese women from Blue Nile State, Sudan.

## Methods

### Study area

A cross-sectional hospital-based study was conducted in the Blue Nile State between January 2012 and December 2015. Blue Nile State lies in the tropical climate zone between latitude 9°30 and 12°30′ and longitude 33°5′ and 35°3 East. Average annual rainfall is around 700 mm with the southern part of the state being the wettest. The State has an area of 45,844 km^2^ and an estimated population of 832,000; 75% of them reside in rural areas, and 25% in the four urban centres. Women represent 47% of the people in the state with maternal mortality rate of 258/100,000 live births. The relevant environmental, anthropological, political unrest and geographic characteristics of Blue Nile State, which shares an international border with Ethiopia and South Sudan, impact uniquely on the epidemiology and control of malaria [17]. The malaria transmission season runs from June in the southern area of the state and July/August to November/December elsewhere.

The study was conducted at the same time in three main maternity units of the hospitals: Ed-Damazin Hospital, El-Roseires Hospital and the Surgical Complex.

The study sites conducted around 6700 deliveries during the study period with the highest number (3968) of deliveries were at the Surgical Complex and the least (789) were at El Roseires Hospital.

Women were recruited after explaining to them the purpose and nature of the study and signing informed consent forms. Women were included irrespective of age, educational background, and socio-economic, cultural or religious condition. Women were approached to participate consented to join the study and to provide the suggested 3 samples (maternal and new born peripheral samples, and placental samples).

### Ethical considerations

The study protocol was discussed and agreed upon initially with health authorities of the Ministry of Health, Blue Nile State prior to being submitted to and reviewed by the Ethical and Scientific Committees of the Tropical Medicine Research Institute and the Directorate of Research, Federal Ministry of Health, Sudan. A signed informed consent was obtained from each woman prior to their enrolment in the study. Women positive for malaria were treated with artesunate-SP according to the National Malaria Control Programme protocol.

### Data collection

Consenting parturient women with singleton pregnancies and normal vaginal were recruited. Pregnancy with twins and mothers with serious delivery complications including hypertension, diabetes mellitus, antepartum haemorrhage or post-partum bleeding, eclampsia and other complicated conditions were excluded. Women were clinically examined and their socio-demographic data, relevant medical and obstetric history were collected. These data were all summarized on a predesigned questionnaire which included information on maternal age, gravidity and gestation, history of previous malaria episodes, use of anti-malarial drugs, IPTp usage and ITNs ownership and use during pregnancy and ANC visits. Birth weight was measured by the attending midwife for all births within 1 h of delivery, using an electronic scale accurate to ±10 grams and calibrated weekly. Newborns were classified as normal birth weight (≥2.5 kg) or LBW (<2.5 kg) according to WHO guidelines [19].

### Sample collection and analysis

At delivery, peripheral and placental blood samples were collected. A finger prick was used to test for malaria microscopy and to measure haemoglobin (Hb) level. Placental blood smears were obtained from the maternal side intervillous space of the placenta using a sterile disposable lancet.

Blood smears were prepared on grease-free, clean glass slides and allowed to dry before being stained with 10% Giemsa at pH 7.2 for 10 min and examined under the ×100 oil immersion objective lens of a light microscope. Microscopic examination of thick films, using high power magnification was used to detect the presence of parasites, while the thin films were used to determine species when thick films were positive. The staining techniques and blood film examination were conducted employing WHO guidelines [20].

Parasite density was determined using the plus system [21], by counting the number of parasites per high power field and rated according to a + scale from + (1–10 parasites per 100 high power fields), ++ (>10 parasite per 100 high power fields), +++ (1–10 parasites in one high power field), to ++++ (>10 parasites in one high power field). If no parasites were seen in 100 fields, the sample was deemed negative.

Maternal haemoglobin concentrations were estimated with a portable HemoCu device (Angelhom, Sweden). Anaemia was defined as Hb < 11 g/dL and Hb < 7 g/dL: severe anaemia, Hb 7–9.9 g/dL: moderate anaemia, and Hb 10–10.9 g/dL: mild anaemia [22].

### Quality control measures

All of the selected research team were centrally trained on the important aspects of the research work and had continuous supervision by the site supervisor. Quality of slide reading was ensured at each of the three study sites then rechecked in Khartoum by an experienced microscopist, blinded to original diagnosis.

### Data analysis

The collected data were analysed using SPSS V.20.0 (SPSS Inc., Chicago, IL, USA). Student’s t test and X^2^ test were used to compare the mean and proportions between two groups, respectively. Binary logistic regressions were built where placental malaria was the dependent variable, the site of samples collection and socio-demographic characteristics were the independent variables. In order to investigate the effect of placental malaria on maternal anaemia and LBW binary logistic regression models were built where these were the independent variables and the site, socio-demographic (age, gravidity, residence, education and ANC visits) and placental malaria were the independent variables. P < 0.05 was considered significant. Parity was categorized as primiparae, secundiparae and multiparae (≥3). For the research purposes, women were diagnosed as malaria positive if parasites were detected by light microscopy in either the peripheral blood or samples collected from the placenta.

## Results

### Characteristics of the study population

A total of 1149 mothers and their newborns fulfilled the inclusion criteria and were had complete data. The mean (SD) of the age was 23.2 (5.2) years. Five hundred and fifty-three (48.1%), 297 (25.8%) and 299 (26.0%) women were primiparae, secundiparae and multiparae, respectively. Less than one-third of the women (30.2%) were rural residents and 922 (80.2%) had education less than secondary level (<9 years). Out of these 1149 women, 452 (39.3%) had antenatal attendance, 102 (8.9%) used bed nets in the index pregnancy and 94 (8.2%) used IPTp.

Five hundred and fifty (47.9%), 227 (19.8%) and 372 (32.4%) women were recruited from the Surgical Complex (549), El Roseires Hospital and Ed-Damazin Hospital, respectively (Table 1).

**Table 1:** Risk factors for placental malaria infection in Sudanese women using univariate and logistic regression analyses

Characteristics	N (%) of the total	Placental positive, N (%)	Univariate analyses		Logistic regression analyses	
			OR (95% CI)	P	OR (95% CI)	P
Site of collection						
Ed-Dmazen Hospital	372 (32.4)	263 (38.8)	2.8 (2.1–3.7)	<0.001	1.1 (0.6–1.8)	0.831
El-Roseires Hospital	227 (19.8)	162 (23.9)	2.9 (2.1–4.1)	<0.001	0.6 (0.4–1.0)	0.051
Surgical complex	550 (47.9)	252 (37.2)	Reference		Reference	
Age groups (years)^a^						
<23.3	674 (58.7)	561 (82.9)	15.3 (11.4–20.5)	<0.001	3.2 (1.9–5.5)	<0.001
≥23.3	475 (41.3)	116 (17.1)	Reference		Reference	
Parity						
Primiparae	553 (48.1)	459 (67.8)	23.1 (15.9–33.6)	<0.001	3.9 (2.1–7.6)	<0.001
Secundiparae	297 (25.8)	166 (24.5)	6.0 (4.1–8.7)	<0.001	2.8 (1.5–5.1)	<0.001
Multiparae	299 (26.0)	52 (7.7)	Reference		Reference	
Residence						
Rural	347 (30.2)	256 (37.8)	2.4 (1.9–3.3)	<0.001	0.7 (0.5–1.2)	0.244
Urban	802 (69.8)	421 (62.2)	Reference		Reference	
Education						
<Secondary level	922 (80.2)	589 (87.0)	2.7 (2.0–3.7)	<0.001	1.7 (1.0–2.8)	0.049
≥Secondary level	227 (19.8)	88 (13.0)	Reference		Reference	
Antenatal care visits						
Not attended	697 (60.7)	566 (83.6)	13.2 (9.9–17.6)	0.001	11.9 (7.8– 18.1)	<0.001
Attended	452 (39.3)	111 (16.4)	Reference		Reference	
Use bed nets						
No	1047 (91.1)	659 (97.3)	2.0 (1.6–2.4)	<0.001	3.5 (1.7–6.8)	<0.001
Yes	102 (8.9)	18 (2.7)	Reference		Reference	
Peripheral malaria						
Positive	434 (37.8)	416 (61.4)	40.2 (24.4–66.0)	<0.001	13.5 (7.6–23.9)	<0.001
Negative	715 (62.2)	261 (38.6)	Reference		Reference	

*N* number, *OR* odds ratio, *CI* confidence interval

^a^Corrected for parity. Four women were missing data on education

### Prevalence of malaria at time of delivery

Based on either peripheral or placental blood smears at delivery, malaria infection was positive in 434 (37.8%) of the smears diagnosed by peripheral blood microscopy, while 677 (58.9%) had placental infection diagnosed by placental blood smear examination. Four hundred and sixteen subjects (36.2%) were both peripheral and placental malaria positive, while 261 of the mothers with negative peripheral blood had placental malaria positive. *Plasmodium falciparum* was the only species detected in all the positive blood smears.

Mild (+) parasitaemia was recorded in 271 (62.4%) and 236 (34.9%) of the positive peripheral blood and placental blood films, respectively, while moderate (++) parasitaemia was found in 142 (32.6%) and 260 (38.4%) of the positive peripheral and placental blood films, respectively. Hyperparasitaemia > +++ was found in 21 (5%) of the maternal peripheral blood and 181 (26.7%) of the placental blood films.

### Factors associated with placental malaria infection

Only 18 women were peripheral smear positive while placental smears negative showing mild (+) parasitaemia and 14 of them (67%) were primiparae. The vast majority of the women (95.9%) who had *Plasmodium falciparum* parasitaemia by microscopy in their peripheral smears at delivery were placental malaria positive.

Younger age ≤23.2 years old (AOR = 3.2, 95% CI 1.9–5.5; *P* < 0.001), primiparae (AOR = 3.9, CI 2.1–7.6; *P* < 0.001), secundiparae (AOR = 2.8, 95% CI 1.5–5.1; *P* < 0.001), no ANC visits (AOR = 11.9, CI 7.8–18.1; *P* < 0.001), not using bed nets (AOR = 3.5, 95% CI 1.7–6.8; P < 0.001) and peripheral blood positivity was significantly associated with placental malaria at delivery (AOR = 13.5, 95% CI 7.6–23.9; *P* < 0.001) were risk factors for placental malaria. Education, residence and site of samples collection were not associated with infection (Table 1).

### Placental malaria and risk factors for adverse pregnancy outcomes

The mean (SD) of the haemoglobin among these women was 10.6 (3.3) g/dL. A high rate of anaemia where 743 (64.7%) women had anaemia, and 22 of them (3%) had severe anaemia (Hb < 7 g/dL). Placental malaria was the highest risk factor for maternal anaemia (AOR = 41.6, 95% CI = 23.3–74.4; P < 0.001) (Table 2).

**Table 2:** Risk factors for maternal anaemia in Sudanese women using univariate and logistic regression analyses

Characteristics	N (%) of the total	Anaemia, N (%)	Univariate analyses		Logistic regression analyses	
			OR (95% CI)	P	OR (95% CI)	P
Site of collection						
Ed-Dmazen Hospital	372 (32.4)	284 (76.3)	2.9 (2.2–3.9)	<0.001	2.0 (1.3–3.3)	0.002
El-Roseires Hospital	227 (19.8)	173 (76.2)	2.9 (2.0–4.1)	<0.001	1.6 (0.9–2.7)	0.074
Surgical complex	550 (47.9)	286 (52.0)	Reference		Reference	
Age groups (years)^a^						
<23.3	674 (58.7)	547 (81.2)	6.1 (4.7–7.9)	<0.001	3.2 (1.6–6.1)	<0.001
≥23.3	475 (41.3)	196 (41.3)	Reference		Reference	
Parity						
Primiparae	553 (48.1)	457 (82.6)	7.8 (5.6–10.8)	<0.001	1.4 (0.7–2.8)	0.252
Secundiparae	297 (25.8)	173 (58.2)	2.2 (1.6–3.1)	<0.001	1.0 (0.5–1.8)	0.874
Multiparae	299 (26.0)	113 (37.8)	Reference		Reference	
Residence						
Rural	347 (30.2)	264 (76.1)	2.1 (1.6–2.8)	<0.001	1.0 (0.6–1.6)	0.955
Urban	802 (69.8)	479 (59.7)	Reference		Reference	
Education						
<Secondary level	922 (80.2)	649 (70.4)	3.3 (2.4–4.5)	<0.001	2.8 (1.6–4.8)	<0.001
≥Secondary level	227 (19.8)	94 (41.4)	Reference		Reference	
Antenatal care visits						
Not attended	697 (60.7)	574 (82.4)	7.8 (5.9–10.2)	<0.001	11.9 (7.8–18.1)	<0.001
Attended	452 (39.3)	169 (37.4)	Reference		Reference	
Use bed nets						
No	1047 (91.1)	702 (67.0)	3.0 (1.9–4.5)	<0.001	1.3 (0.8– 2.4)	0.228
Yes	102 (8.9)	41 (40.2)	Reference		Reference	
Peripheral malaria						
Positive	434 (37.8)	417 (96.1)	29.2 (17.6–48.5)	<0.001	6.6 (3.4–12.5)	<0.001
Negative	715 (62.2)	326 (45.6)	Reference		Reference	
Placental malaria						
Positive	677 (58.9)	639 (94.4)	59.5 (40.1–88.1)	<0.001	41.6 (23.3–74.4)	<0.001
Negative	472 (41.1)	104 (22.0)	Reference		Reference	

*N* number, *OR* odds ratio, *CI* confidence interval

^a^Corrected for the site. Four women were missing data on education

The overall mean (SD) of the birth weight of the neonates was 2.5 (0.38) kg and the overall frequency of LBW was 56.4% (n = 684). Malaria infection was significantly associated with LBW with the mean (SD) birth weight in malaria negative women being 2.8 (0.33) kg while that in peripheral malaria positive women was 2.3 (0.20) kg and in placental malaria positive women was 2.2 kg (0.20 kg). Maternal anaemia (AOR = 6.5, 95% CI 3.9–10.8; *P* < 0.001), placental malaria (AOR = 25.02, 95% CI 15.1–41.3; *P* < 0.001), and no ANC visits (AOR = 1.7, CI 1.1–2.7; *P* = 0.010) were significant risk factors for low birth weight (Table 3).

**Table 3:** Risk factors for low birth weight in Sudanese women using univariate and logistic regression analyses

Characteristics	N (%) of the total	Low BW, N (%)	Univariate analyses		Logistic regression analyses	
			OR (95% CI)	P	OR (95% CI)	P
Site of collection						
Ed-Dmazen Hospital	372 (32.4)	248 (38.2)	2.4 (1.8–3.2)	<0.001	1.1 (0.6–1.7)	0.644
El-Roseires Hospital	227 (19.8)	156 (24.0)	2.7 (1.9–3.7)	<0.001	1.3 (0.7–2.2)	0.305
Surgical complex	550 (47.9)	245 (37.8)	Reference		Reference	
Age groups (years)						
<23.3	674 (58.7)	522 (80.4)	9.4 (7.1–12.3)	<0.001	1.1 (0.6–2.1)	0.598
≥23.3	475 (41.3)	127 (19.6)	Reference		Reference	
Parity						
Primiparae	553 (48.1)	432 (66.6)	12.1 (8.6–16.9)	<0.001	1.0 (0.5–2.1)	0.840
Secundiparae	297 (25.8)	149 (23.0)	3.4 (2.4–4.8)		0.7 (0.4–1.4)	0.424
Multiparae	299 (26.0)	68 (10.5)	Reference	<0.001	Reference	
Residence						
Rural	347 (30.2)	245 (37.8)	2.3 (1.8–3.0)	<0.001	1.5 (0.5–1.2)	0.300
Urban	802 (69.8)	404 (62.2)	Reference		Reference	
Education						
<Secondary level	922 (80.2)	567 (87.4)	2.8 (2.0–3.8)	<0.001	1.5 (0.8–2.8)	0.153
≥Secondary level	227 (19.8)	82 (12.6)	Reference		Reference	
Antenatal care visits						
Not attended	697 (60.7)	531 (81.8)	9.0 (6.8–11.8)	<0.001	1.7 (1.1–2.7)	0.010
Attended	452 (39.3)	118 (18.2)	Reference		Reference	
Use bed nets						
No	1047 (91.1)	622 (95.8)	4.0 (2.5–6.4)	<0.001	1.5 (0.8–3.0)	0.161
Yes	102 (8.9)	27 (4.2)	Reference		Reference	
Anaemia						
Yes	743 (64.7)	611 (94.1)	44.8 (30.5–65.7)	<0.001	6.5 (3.9–10.8)	<0.001
No	406 (35.3)	38 (5.9)	Reference		Reference	
Peripheral malaria						
Positive	434 (37.8)	404 (62.2)	25.8 (17.2–38.6)	<0.001	5.5 (3.3–9.1)	<0.001
Negative	715 (62.2)	245 (37.8)	Reference		Reference	
Placental malaria						
Positive	677 (58.9)	601 (92.6)	69.8 (47.6–102.3)	<0.001	25.0 (15.1–41.3)	<0.001
Negative	472 (41.1)	48 (7.4)	Reference		Reference	

*N* number, *OR* odds ratio, *CI* confidence interval

Four women were missing data on education

## Discussion

The present study determined the prevalence of placental malaria among women delivering at the main hospitals in Blue Nile State, Sudan to be 58.9%. This is lower than the prevalence reported elsewhere [23, 24], and was higher than the prevalence of placental infections for women of all gravidities, ranging from 5 to 52% that had been reported in many African countries [25]. The factors responsible for the variations in prevalence were reported to be acquired immunity related to the malaria transmission in the various setting [26, 27]. A potential reason of the high prevalence in this study is that no enhanced control activities such as two prevention approaches advocated by the WHO [18]. In Afghanistan, it has been reported that avoidance of medication during pregnancy and intermittent preventive treatment was hard to justify or implement and preventive strategy should instead focus on long-lasting insecticidal nets for all pregnant women [28]. However, it has been recommended to scale up malaria prevention efforts in more isolated and deprived communities as highlighted in a meta-analysis of datasets from 25 African countries [29]. Nevertheless, Cisse and colleagues [7] reported that the use of IPTp-SP does not reduce the risk of malaria incidence during pregnancy.

The findings of this study were in accordance with those of Uneke [30], who found that maternal peripheral malaria may range in prevalence from 9 to 60% in the malarious areas of sub-Saharan Africa. Placental parasitaemia without peripheral parasitaemia was observed in the present study. It is recognized that peripheral parasitaemia may remain below the levels of microscopic detection although parasites harbored the placenta [24]. Moreover, low number of peripheral parasitaemia without placental infection was also found and may occur in early malaria infection, especially if parasitaemia is low [31]. The present study confirmed a significant association of being primiparae (AOR = 1.371, CI (1.82–2.28; *P* = 0.012), and young age (AOR = 5.79, CI 11.91–17.60; *P* = 0.002), with placental malaria. This finding is consistent with those of Brabin [5]; Bouyou-Akotet et al. [32] and Taco et al. [9] who reported young primigravidae were more likely to have placental malaria. Development of the immunity to the placenta-binding parasites e.g. production of antibodies that inhibit the adherence of placental parasites to CSA acquired with subsequent pregnancies [33, 34]. A correlation of borderline significance was found between education and placental malaria (*P* = 0.049). Poor women from rural areas in the study area usually deliver at home and tend to be less aware of the need to avoid infection because most of the education programmes are concentrated in the urban areas. This factor must be an important driver towards the design and implementation of programmes at community level in rural areas. Ezebialu et al. [24] reported that rural poor women, who are more likely to be infected with malaria, are more likely to attend maternity homes and smaller private clinics.

In consistent with previous studies, the present study showed that neonates of mothers with placental malaria were LBW [4, 25, 35], while Adam et al. documented no association between placental malaria and LBW in eastern Sudan [36, 37]. Importantly, the present study found that peripheral malaria infection at delivery was also associated with lower birth weight. This consistent with another study carried out in Africa [4].

In this study, there was an association between placental malaria and maternal anaemia similar to previously published studies from several countries in sub-Saharan Africa which indicated that malaria infected women had higher prevalence of anaemia than uninfected women [9, 38]. The causes of anaemia in pregnancy are multi-factorial [39] and poor nutrition and poverty are direct causes of anaemia.

### Limitations of the study

The study extended from 2012 to 2015 and samples were collected in order to investigate the prevalence of placental malaria at the study sites. This extended duration was due to availability of field expenses and resources. Moreover, recruitment and samples collection was not carried on a daily basis due to the limited number of midwives participated in the study. Parasite density was the existing routine practice in the laboratories of the study sites as the technicians participated the study were not researchers.

## Conclusion

There was a high prevalence of placental malaria in this part of Sudan especially younger age and primiparae. Placental malaria was associated with maternal anaemia and LBW.

### Recommendations

Community based rigorous education programme to increase awareness of the importance of ANC services and the safety of anti-malarial drugs during pregnancy should be encouraged among all pregnant women, especially the primigravidae. An assessment of the control measures available in the area particularly providing free ITNs should be urgently implemented. Further community based studies should follow.
